# Teaching through technology: comparing student experiences of VR and computer simulations in orthoptics education

**DOI:** 10.1186/s42234-026-00214-y

**Published:** 2026-08-29

**Authors:** Michael Batterley, Georg Meyer, Jignasa Mehta, Simon Campion, Ryan Ward

**Affiliations:** 1 University of Liverpool, Liverpool, UK; 2 Department of Computer Science & Mathematics, Liverpool John Moores University, Liverpool, UK

**Keywords:** Higher Education, Learning Environment, Orthoptics, Students, Virtual Reality

## Abstract

**Background:**

Virtual Reality (VR) has previously been found to provide both an effective and enjoyable learning environment for users to develop a wide range of skills. However, despite uptake in fields such as medicine and veterinary medicine, there is little research into the use of VR for diagnostics training with Orthoptists.

**Methods:**

This study compared students’ experiences of a collaborative VR learning environment with the Strabismus desktop computer simulation for practicing the diagnosis of ocular deviations and limitations. The VR system provided stereoscopic depth and natural controller-based interaction, enabling students to practice diagnostic procedures with greater spatial realism than 2D simulators. Thirty one first year and twenty one second year Orthoptics students from the University of Liverpool were tasked with diagnosing simulated patients in both learning environments. After completing their diagnoses, students’ user experience, sense of presence, comfort with patient proximity, and their thoughts on the use of VR in higher education (SHEQ) were measured.

**Results:**

Students reported higher hedonic user experience scores for VR, whereas pragmatic user experience scores were higher for the desktop simulation. SHEQ responses indicated a general preference for VR as a learning tool. However, there were no significant differences in sense of presence, or comfort with proximity to patient scores.

**Conclusions:**

This study shows how commodity VR hardware can be adapted for specialist healthcare training. Although VR did not demonstrate advantages across all measured outcomes, students generally perceived it positively and preferred it over using 2D displays as a learning tool within higher education. These findings suggest that VR may have potential as a supplementary educational resource for Orthoptics training. However, further research is required to determine whether these positive perceptions translate into real world improvements in learning, diagnostic performance, and clinical practice.

## Introduction

Orthoptics is an allied health profession specialising in the diagnosis and treatment of defects in eye movements (British and Irish Orthoptic Society n.d.). Orthoptists play an important role in performing patient examinations and diagnosing a variety of eye disorders which include strabismus (eye misalignment), ocular deviations, and limitations in ocular motility (Pritchard 2016; Mayo Clinic College of Medicine & Science n.d.). Given the complexities and precision involved in these diagnostic tasks, inaccuracy or failure to provide appropriate treatment could result in compromised visual health, as well as a reduced quality of life for the patient. Therefore, Orthoptists require extensive university training in the form of university lectures combined with clinical placements to educate students on a variety of different eye conditions and interventions (University of Liverpool 2025; University of Sheffield 2025; British and Irish Orthoptic Society n.d.). These clinical placements are to provide student orthoptists a learning environment whereby they apply their theoretical knowledge to real world patients under the direct supervision of a qualified Orthoptist.

Placement capacity is a challenge due to the increase in student numbers and their effectiveness being dependent on the availability of patients, concern for patient safety and the exposure of students to a range of different eye conditions. Students are expected to be exposed to different types of strabismus depending on the level they are at within the curriculum, and placement sites cannot consistently guarantee this learning experience. Furthermore, with only 5% of the population suffering from binocular vision disorders, and 74% of these disorders being strabismus (Stidwill 1997), it makes it difficult for students to experience a broad range of symptom severity for a specific or rare eye disorder. Alternative virtual learning environments (VLEs) could be used to enable students to observe a much wider range of eye conditions while avoiding many of the associated risks.

VLEs have the potential to provide Orthoptics students with a space to apply their theoretical understanding to accurately diagnose virtual patients. For example, the Strabismus simulator (Faruk and Orge 2015) is a 2D interactive computer simulation which allows orthoptists to practice basic strabismus evaluations. This includes, the cover-uncover test, alternate cover test, alternate cover test with prism, and simultaneous prism cover test. The first benefit of using this simulator for training is that it enables the user to access the practice material on any desktop computer, at any time, and for as long as they want without the need for a supervisor and a human patient being present. Secondly, as the simulation uses virtual patients, students are able to develop their diagnostics skills in a safe learning environment, without risk to patients’ health. Finally, using the pre-programmed simulations students are able to practice diagnosing patients with a wide range of eye disorders which they may not be exposed to during their clinical placements.

Whilst computer-based simulations can be effective at improving learning outcomes (Musa 2022; D’Angelo et al. 2014; Rutten et al. 2012; Seymour et al. 2002), learning on a 2D display does have some limitations. During a cover-test for example, an Orthoptist will use varying aspects of their sense of sight, sound and touch to come to a diagnosis. This includes using their sight to judge the distance of the ocular occluder to the patient’s face, sound when verbally instructing the patient to follow the examination torch, and touch to handle the various pieces of equipment. However, performing this task using a 2D display would prove difficult. In the Strabismus simulator for example, the user operates a mouse to drag and drop three pieces of equipment (a transparent occluder, fixation target and prism) to come to a diagnosis. As a computer screen is monoscopic (displaying only one image), it would be more difficult to establish a sense of depth compared to the stereoscopic view (displaying two different images) that people experience in the real world (Wenk et al. 2023; Mikkola et al. 2012). Given the development of motor control skills, such as reaching for an object or using diagnostic equipment, require the use of depth perception (Kato et al. 2016), learning using a 2D display would result in the restriction of key motor skills development.

Virtual Reality (VR) has been found to be an effective learning environment across a wide range of topic areas (Batterley and Limniou 2026). This includes language learning (Peixoto et al. 2021), training first responders for mass casualties (Kman et al. 2023), motor skills training in sports (Michalski et al. 2019), and developing students’ employability skills (Batterley et al. 2025). Whilst similar in many ways to a computer environment, VR has many of the benefits of using a computer-based simulation but is also able to address some of the limitations. For example, handheld VR controllers allow the user to interact with objects in the simulated environment. In addition to VR being able to develop users’ motor skills (Juliano and Liew 2020), the natural movement of using a VR controller has been found to increase the user’s sense of presence (Seibert and Shafer 2018). This is particularly important as sense of presence has been linked to improved learning outcomes (Wei et al. 2025; Lin et al. 2023).

The process of diagnosing eye conditions often requires the orthoptist to be within close proximity of the patient. This physical closeness can be problematic, as people may report discomfort when positioned within one metre of another person (Kroczek et al. 2020). Students undertake placements throughout the three year program. Whilst second year students who have already undertaken a clinical placement may not feel this discomfort, inexperienced first year orthoptic students may not feel comfortable in close proximity during eye examinations. VR may help address this issue by simulating the physical arrangement of an orthoptic examination, allowing students to repeatedly practice interacting with virtual patients in a safe and controlled environment prior to clinical placement. Repeated exposure to these simulated clinical interactions may help students become more familiar with the examination process and reduce uncertainty associated with working in close proximity to patients. Exposure based rehearsal has also been associated with improvements in confidence during VR training (Sinurat et al. 2025). Therefore, training orthoptists in a VR environment may improve students’ perceived comfort with conducting examinations in close proximity to real-world patients.

Whilst VR has the potential to provide orthoptics students with a useful learning environment, research into this area is limited. Therefore, this study compares VR and desktop computer learning environments for helping Orthoptists practice the diagnosis of eye conditions in virtual patients. This study has the following five hypotheses to determine whether VR offers a better experience than the desktop computer condition:

1. Pragmatic User experience will be significantly greater in the VR condition compared to the desktop computer condition
2. Hedonic User experience will be significantly greater in the VR condition compared to the desktop computer condition
3. There will be a significantly greater sense of presence in the VR condition compared to the desktop computer condition
4. Students will be significantly more comfortable with being in close proximity to real-world patients following the VR condition compared to the desktop computer condition
5. Students will have a greater preference for using VR as a form of learning in higher education compared to using a 2D display

## Materials and methods

### Experimental design

The study used a counterbalanced within-subjects design with the learning environment (VR versus desktop computer) as the independent variable. Participants were randomly allocated into groups of three based on scheduled testing sessions. Counterbalancing was implemented at the group level by alternating the order of the learning environment conditions across testing sessions. The dependent variables were user experience, sense of presence, comfort with proximity to a real-world patient, and students’ thoughts of VR in higher education.

### Virtual reality setup

Three learning stations were created with an HTC Vive Focus 3 and an accompanying laptop at each station. The laptops used had an i7-11800H 2.3 GHz CPU, 32GB RAM and a 16GB NVIDIA GeForce RTX 3080 GPU.

**Fig. 1:**
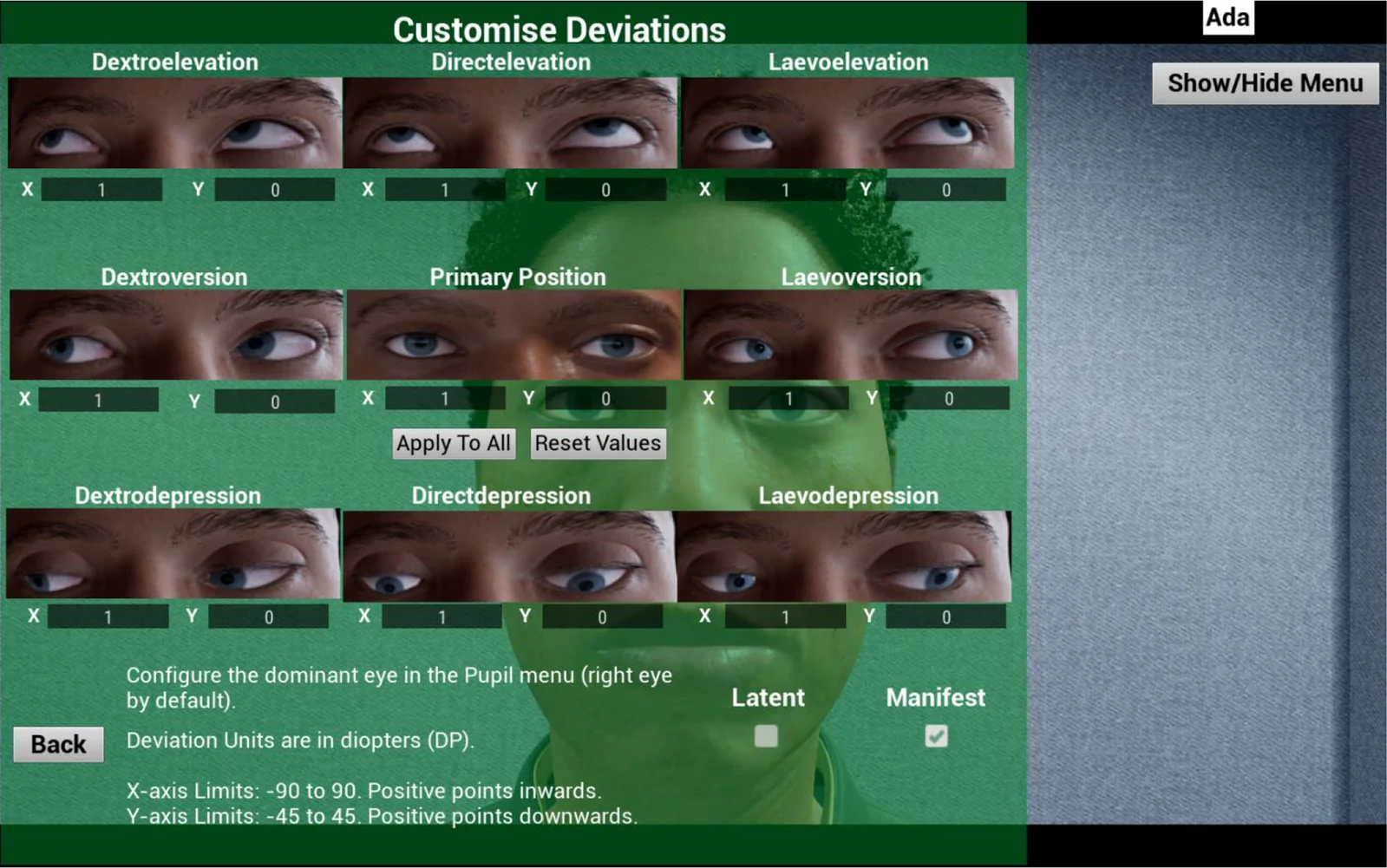
VR software setup of the virtual patient with the above eye deviations and manifest

**Fig. 2:**
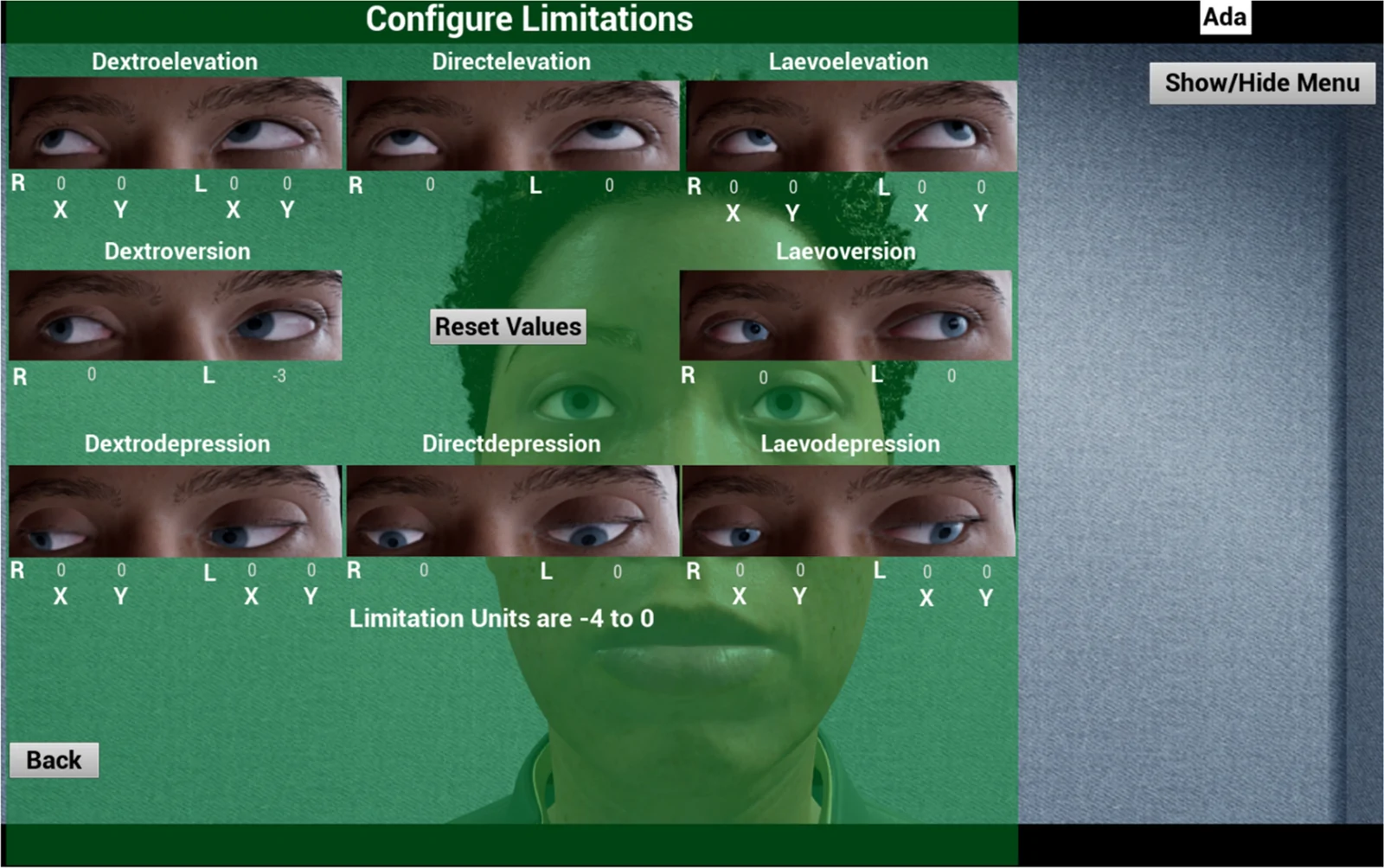
VR software setup of the virtual patient with the above eye limitations

**Fig. 3:**
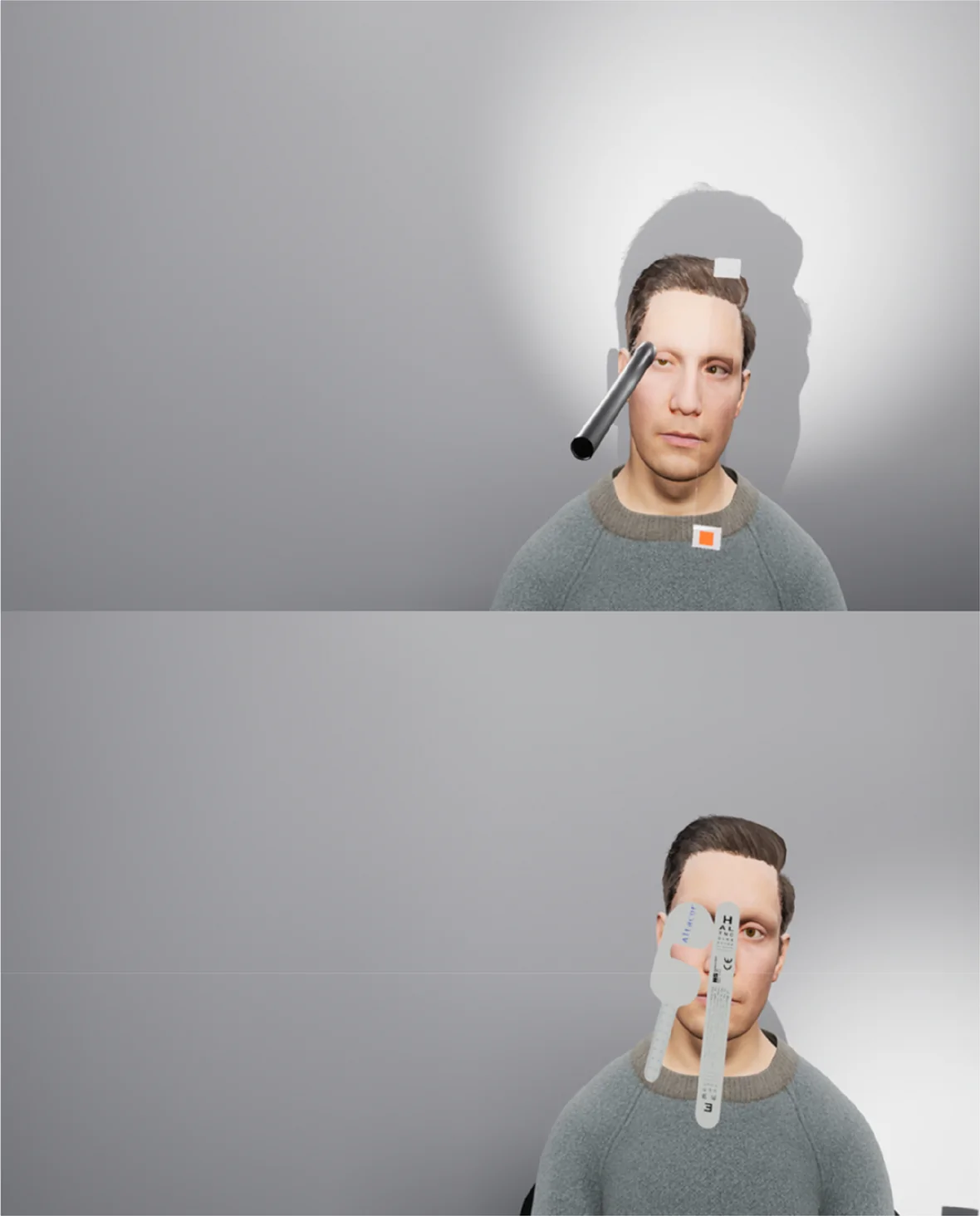
Screenshot of a virtual patient inside the VR simulation

The students were tasked with diagnosing eye deviations and limitations for a 3D virtual patient which were preset using the simulation. The virtual patient details on this simulation were as follows (See Fig. 1 for the setup of the patients’ eye deviations, Fig. 2 for the setup of the patients’ eye limitations, and Fig. 3 for an example of a virtual patient inside the VR simulation):

1. Deviation – all positions on the x-axis had a + 1 deviation on the left eye in all positions
2. Limitation – looking to the right in the left eye, the movement was limited to only 25% of the normal movement
3. The patient’s condition was manifest, i.e., a visible misalignment of the eye

### Desktop computer web-based simulation setup

**Fig. 4:**
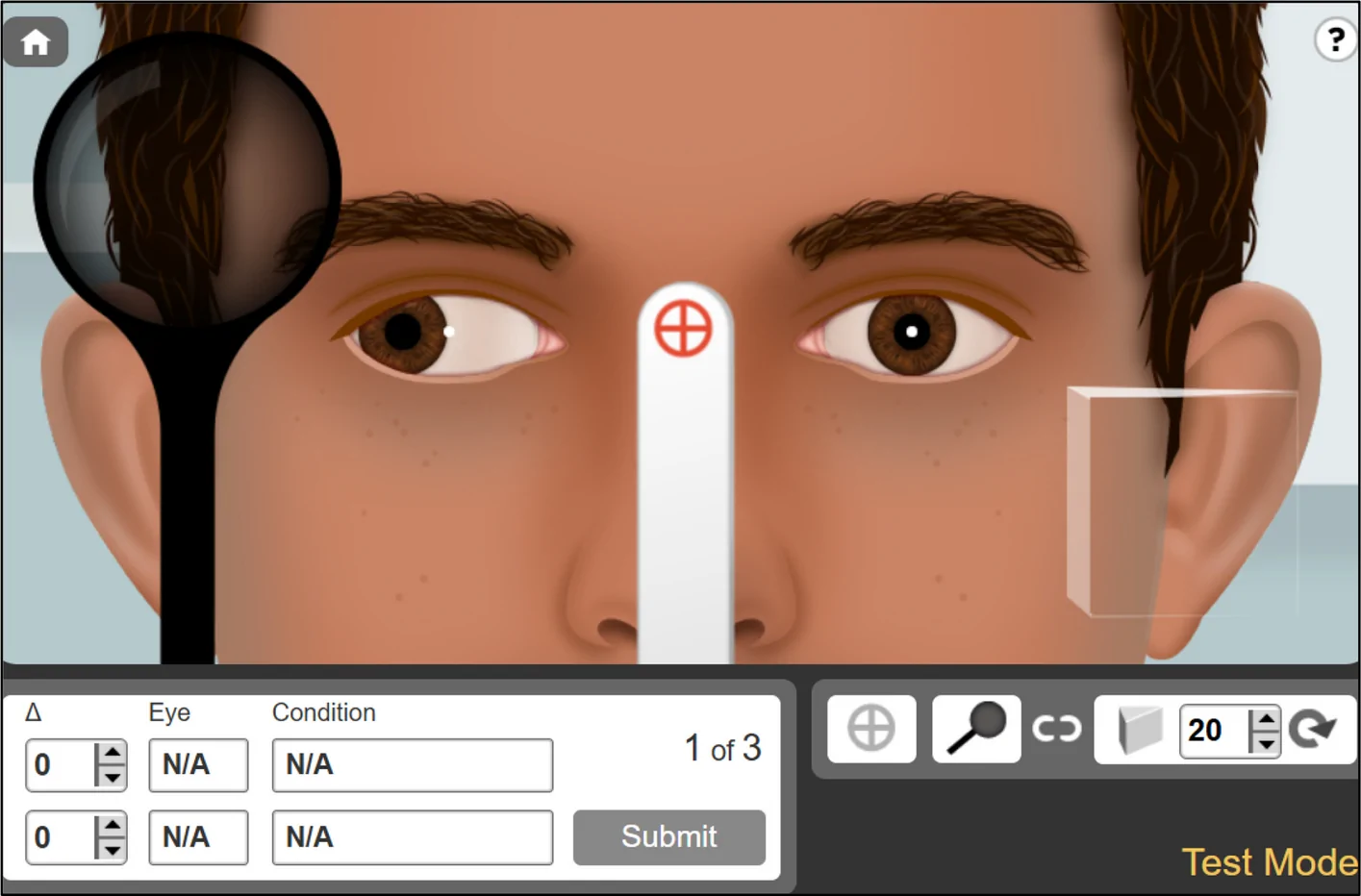
Screenshot of the strabismus simulation

For this task, students remained in the same groups as in the VR task. Each group used the same laptop as the VR condition to diagnose three separate 2D virtual patients. The simulation used in this condition was the Strabismus Simulator developed by Faruk and Orge (2015). A link to the simulation can be found in Fig. 4. Students were tasked with working as a group to diagnose eye deviations and limitations for three different virtual patients. Virtual patients’ conditions were randomised for each group of students. Upon completion of all diagnoses, students were then presented with the correct diagnosis for each patient (Fig. 4 illustrates the strabismus simulation).

The Strabismus Simulation can be found at https://www.aao.org/education/interactive-tool/strabismus-simulator

### Questionnaires

#### Comfort with proximity to patients questionnaire

This questionnaire contains 10 items and was developed by the researchers to measure students’ comfort with being in close proximity to a patient in the real world. Participants rated their comfort on a 7-point Likert scale ranging from 1 = Strongly disagree to 7 = Strongly agree. The questionnaire included both positively and negatively worded items to reduce acquiescence bias. An example of a positively worded item was “*I would feel comfortable being able to use equipment near the patient’s face*”, whereas a negatively worded item was “*I would feel uncomfortable when the patient and I make eye contact*”. Higher scores on the questionnaire indicate a greater perceived comfort with being in close proximity to a patient. See Appendix A for a copy of the questionnaire.

#### User experience questionnaire short (UEQ-S)

The UEQ-S is an 8-item questionnaire developed by Schrepp et al. (2017) to assess the user’s experience of the simulation using a 7-point Likert scale between two different descriptors, e.g., “Clear” and “Confusing”. The questionnaire has two components: pragmatic and hedonic. Pragmatic items measure interaction qualities that relate to the tasks or goals the user aims to reach when using the simulation. Hedonic items on the other hand do not measure tasks and goals, but instead measure aspects related to pleasure or fun whilst using the simulation. See Appendix B for a copy of the questionnaire.

#### Sense of presence

An abridged version of the Witmer and Singer (1998) (see Appendix C) presence questionnaire was used to measure how much the user is experiencing the simulated environment as opposed to the physical world. Participants’ sense of presence was measured using questions such as “*How natural did your interactions with the simulation seem*?”.

#### Students in higher education questionnaire (SHEQ)

The SHEQ developed by Cicek et al. (2021) (see Appendix D) assesses preference for VR over a 2D display, whether VR would increase interest in teaching content, and whether introduction of VR in education could improve learning outcomes. Participants responded to the questions on a scale from 1 (completely disagree) to 5 (completely agree). An example question in this questionnaire is “*Due to the simulation and experience provided by VR, students will continue to explore and research the educational content*”.

### Procedure

After obtaining informed consent, students were organised into groups of three people per station due to time constraints and the limited availability of VR equipment. Following completion of the demographics questionnaire, students took part in either the VR or the computer condition for ten minutes. During the VR condition, the student making the diagnosis was placed in the VR setup whilst the remaining group members observed the procedure using the laptop screen. Students watching the simulation on the laptop were encouraged to communicate with the student in VR to assist them in coming to a diagnosis. Due to time limitations with the students and the time required to set the participant up in the VR equipment, only one virtual patient was presented for diagnosis. The student using the VR headset was selected on a voluntary basis. The identity of the students who used the VR headset was not recorded, meaning that it was not possible to determine how many of the participants directly experienced the simulation through the headset versus observing the simulation on the laptop. Roles were not rotated due to the limited time available for each testing session and the additional setup and calibration time required for each participant using the VR equipment. Group members were encouraged to communicate and assist with the diagnosis in both the VR and computer conditions to replicate the collaborative discussions and peer supported learning commonly observed within Orthoptics education and clinical training environments. After they finished the relevant task or ten minutes had elapsed, students then completed the respective comfort with proximity to patients, UEQ-S, and sense of presence questionnaires individually. Following this, students took part in the other learning environment condition for ten minutes. The same questionnaires in the first condition were then completed. Students then filled out the SHEQ. They were then presented with an open text response box asking for feedback regarding the learning environments that they have taken part in before being debriefed. The study took approximately 30 min to complete.

### Participants

To determine the required sample size for this study, a power analysis was conducted using G*Power (Faul et al. 2009). VR training has previously been found to elicit large effect sizes (Batterley and Meyer 2025; Akman and Çakır 2023; Buttussi and Chittaro 2020; Zając-Lamparska et al. 2019). For a more conservative approach, the analysis was conducted with a medium effect size (Cohen’s d of 0.5), a power of 0.9 and 2 groups (VR and computer learning environment) for a within subjects design. This analysis indicated that a total of 44 participants were required to take part in the study. A total of 67 undergraduate Orthoptics students took part in the study; 37 of these were first year students with the remaining 30 of being second year students. However, due to the group nature of the study, a large number of students worked on the questionnaire as a group despite being told to do this individually. This resulted in 15 participants being removed from the analysis, leaving 52 participants (31 first years, 21 second years) with useable data.

To be eligible to take part in the study, participants needed to be undergraduate students studying Orthoptics at the University of Liverpool, over the age of 18, and have normal or corrected to normal (e.g., glasses) vision and hearing. Students were recruited through opportunity sampling from the Orthoptic programme at the University of Liverpool.

### Questionnaire scoring and statistical analyses

For the UEQ-S, pragmatic and hedonic scores were calculated separately in accordance with the scoring guidelines proposed by Schrepp et al. (2017). Higher scores indicated a more positive user experience. Presence scores were calculated by taking the mean score across all items in the abridged Witmer and Singer (1998) presence questionnaire, with higher scores indicating a greater sense of presence within the simulated learning environment.

The SHEQ responses were analysed across three components. The first component examined students’ preferences for learning using a HMD VR system compared with a traditional 2D display. The second component investigated whether students believed VR would increase interest in educational content. This included students’ opinions regarding the use of VR both inside and outside of the classroom for educational purposes, the importance of the social aspect of the educational process, and whether VR improved students’ understanding of the teaching content. The third component examined whether students believed that integrating VR into the higher education curriculum would improve learning outcomes. This included perceptions of the efficiency of the learning process, the educational system and methods of student evaluation, and the integration of VR into education. Percentages of agreement, disagreement, and neutral responses were calculated for each component.

For the comfort with patient proximity questionnaire, responses were coded from 1 (Strongly disagree) to 7 (Strongly agree). Negatively worded items were reverse scored prior to analysis. These negative items included items 1, 3, 4, 6, and 9. Reverse scoring was performed by subtracting the original response from eight (i.e., a score of 1 became 7, a score of 2 became 6, etc.). Following reverse scoring, an overall mean comfort score was calculated by averaging responses across all questionnaire items, so that higher scores reflected a greater perceived comfort with being in close proximity to patients.

**Fig. 5:**
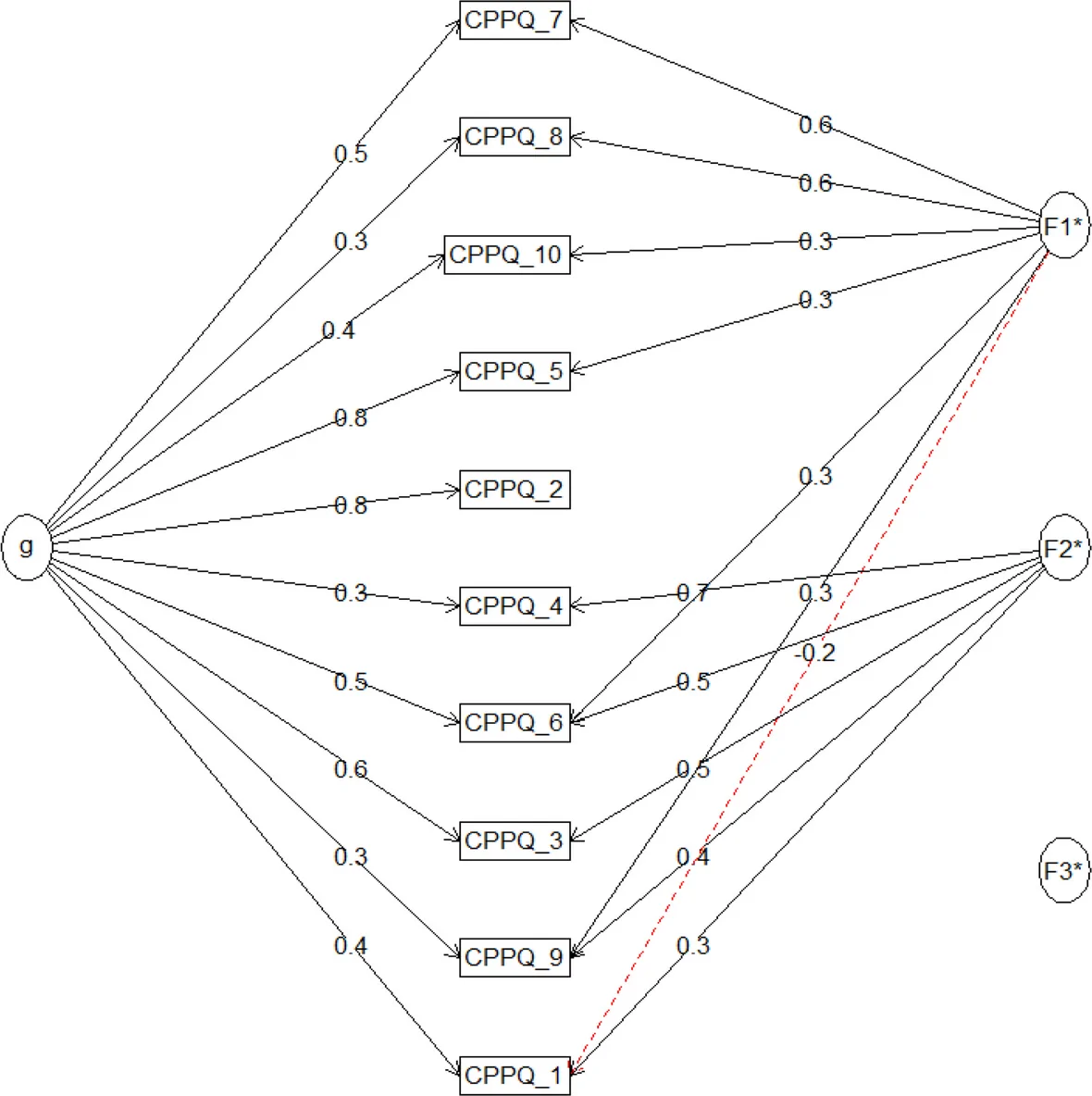
Factor loadings for each item in the comfort proximity to patient questionnaire

As the comfort with patient proximity questionnaire was developed by the researchers, the internal consistency of the questionnaire was first assessed using McDonald’s Omega. An Omega hierarchical (ωh = .62), suggested 62% of the variance in comfort with patient proximity scores were due to a general factor, indicating the potential presence of multidimensionality within the scale. However, the Omega total (ωt = .87) indicated good overall internal consistency across the questionnaire items. Given that the questionnaire was designed to assess the broader construct of comfort with patient proximity, and considering the exploratory nature of this newly developed measure, the overall mean score across all the items was retained for the analyses. Previous psychometric research has suggested that questionnaires intended to measure a broad general construct may still demonstrate some multidimensionality whilst retaining a meaningful overarching factor across the items (Flora 2020). Therefore, despite the moderate omega hierarchical value, the high omega total supported the use of an overall score as a general measure of comfort with patient proximity. Based on these findings, each of the following three analyses were based on the overall mean scores across all of the questionnaire items. The greater the score on the comfort questionnaire, the more comfortable they felt with being in close proximity to real world patients. For a copy of the individual item factor loadings, see Fig. 5.

The CPPQ represents the Comfort with Proximity to Patients Questionnaire, with the number following this identifying the question number. For example, CPPQ _3 would represent the third question in this questionnaire.

Linear mixed effects models were conducted to investigate the effects of learning environment and year group on UEQ-S pragmatic scores, UEQ-S hedonic scores, presence scores, and comfort with proximity to patient scores. Post-hoc comparisons were conducted where appropriate. Partial eta squared effect sizes are reported for all analyses.

All statistical analyses were conducted in R (version 4.4.1) using the lme4 and lmerTest packages. Separate models were fitted for UEQ-S pragmatic, UEQ-S hedonic, sense of presence, and comfort with patient proximity scores. Learning environment, year group, and their interaction were entered as fixed effects. Participant was included as a random intercept to account for the repeated measures design. Group identifiers and condition order were not retained in the final dataset, therefore could not be included as additional random and fixed effects. Models were fitted using restricted maximum likelihood (REML) estimation via the default lmer() procedure. Statistical significance was assessed using the F-tests from the lmerTest package, with Satterthwaite’s approximation used to estimate degrees of freedom. Where appropriate, pairwise comparisons were conducted using estimated marginal means from the emmeans package. Partial eta-squared effect sizes were calculated for each model.

## Results

### UEQ-S pragmatic

The linear mixed effects model indicated that there was a significant main effect of learning environment on UEQ-S pragmatic scores, F(1, 35.36) = 7.38, *p* = 0.008, np^2^ = 0.18. The computer learning environment (mean = 0.83, SD = 1.14) produced significantly greater user experience pragmatic scores than the VR learning environment (mean = 0.14, SD = 1.38).

There was no significant main effect of year group on UEQ-S pragmatic scores, F(1, 41.18) = 0.13, *p* = 0.720, np^2^ = 0.03. The year one group (mean = 0.43, SD = 1.33) did not produce significantly greater user experience pragmatic scores compared to the year two group (mean = 0.55, SD = 1.30).

Finally, no significant learning environment interaction was found, F(1, 35.36) = 0.57, *p* = 0.450, np^2^ = 0.02.

**Fig. 6:**
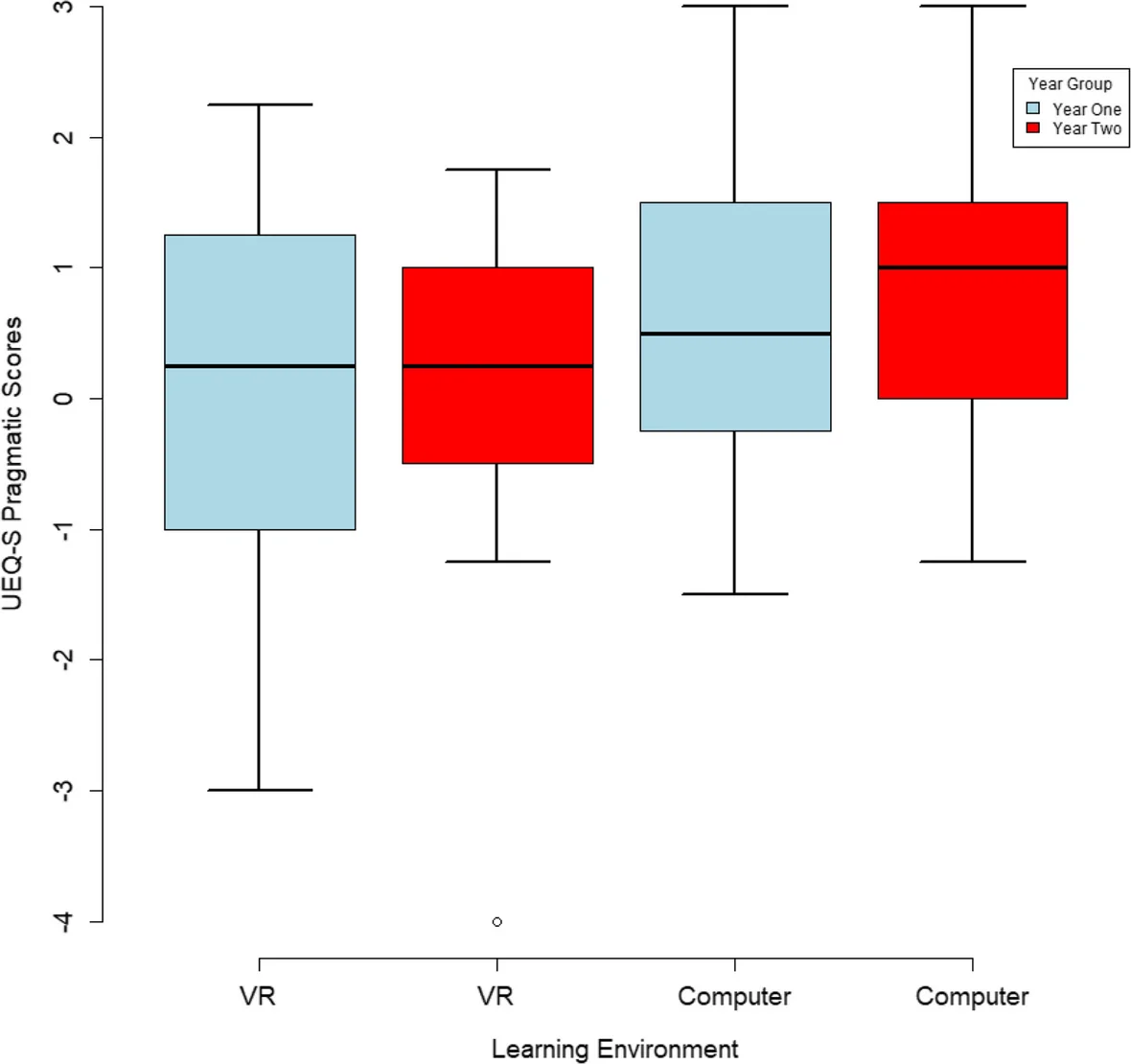
Boxplots comparing UEQ-S pragmatic scores across learning environment and year group

**Fig. 7:**
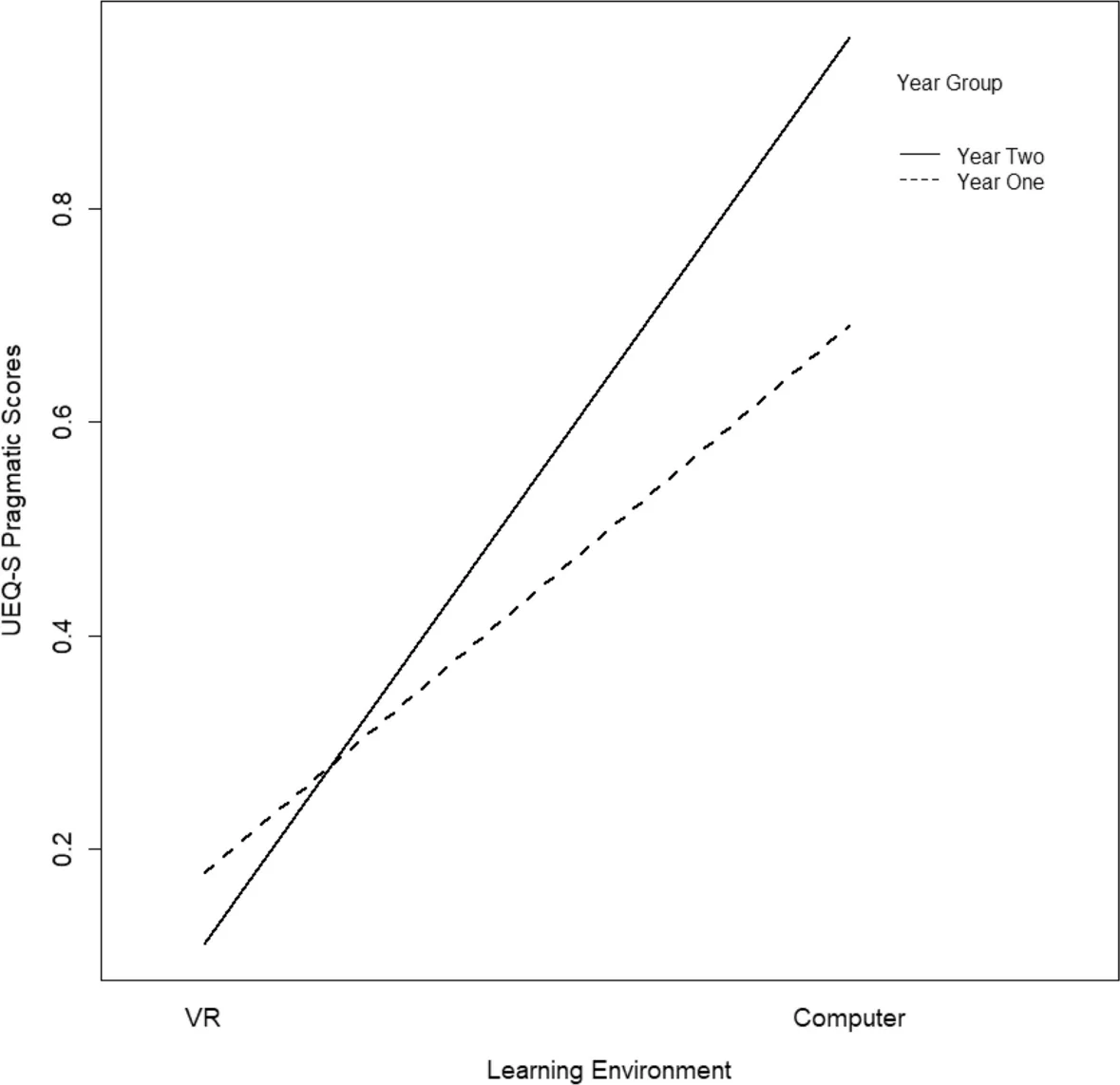
Interaction plot for learning environment and year group on UEQ-S pragmatic scores

A boxplot of the UEQ-S pragmatic scores across learning environment and year group can be found in Fig. 6. The interaction plots for learning environment and year group on UEQ-S pragmatic scores can be found in Fig. 7. A copy of the descriptive statistics for the UEQ-S pragmatic scores across the VR and computer conditions split by year group can be found in Table 1.

**Table 1:** Descriptive statistics for UEQ-S pragmatic scores in VR and Desktop conditions split by year group

	Mean (± SD)		
	VR UEQ-S Pragmatic Score	Computer UEQ-S Pragmatic Score	Total
Year 1	0.18 (1.44)	0.69 (1.19)	0.43 (1.33)
Year 2	0.11 (1.36)	0.96 (1.12)	0.55 (1.30)
Total	0.14 (1.38)	0.83 (1.14)	

### UEQ-S hedonic

The linear mixed effects model indicated that there was a significant main effect of learning environment on UEQ-S hedonic scores, F(1, 30.02) = 7.08, *p* = 0.012, np^2^ = 0.19. The VR learning environment (mean = 1.39, SD = 1.14) produced significantly greater user experience hedonic scores than the computer learning environment (mean = 0.97, SD = 1.29).

There was no significant main effect of year group on UEQ-S hedonic scores, F(1, 40.19) = 1.15, *p* = 0.290, np^2^ = 0.03. The year one group (mean = 0.97, SD = 1.05) did not produce significantly greater user experience hedonic scores compared to the year two group (mean = 1.36, SD = 1.36).

Finally, no significant learning environment interaction was found, F(1, 30.02) = 0.27, *p* = 0.604, np^2^ < 0.01.

**Fig. 8:**
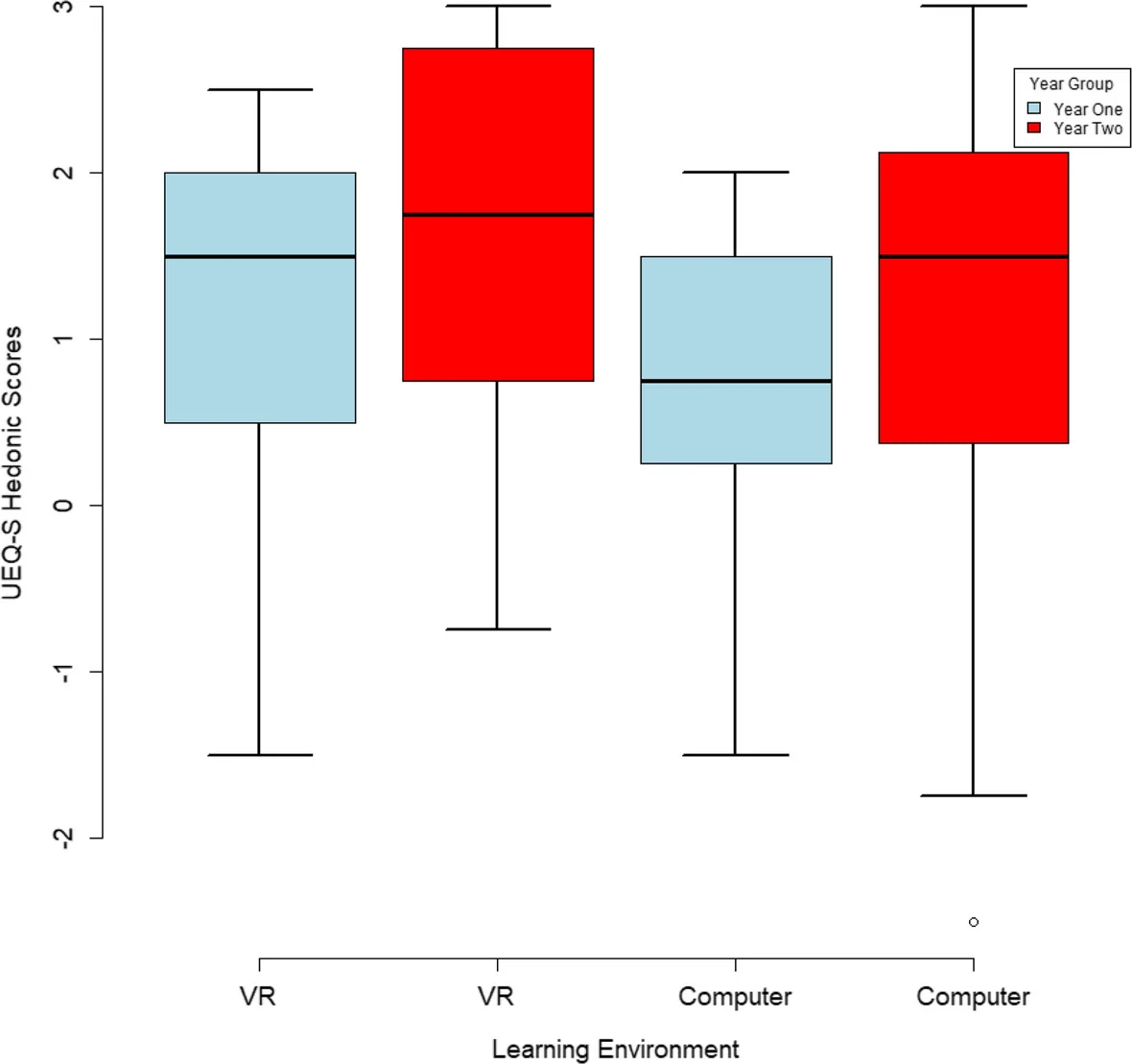
Boxplots comparing UEQ-S hedonic scores across learning environment and year group

**Fig. 9:**
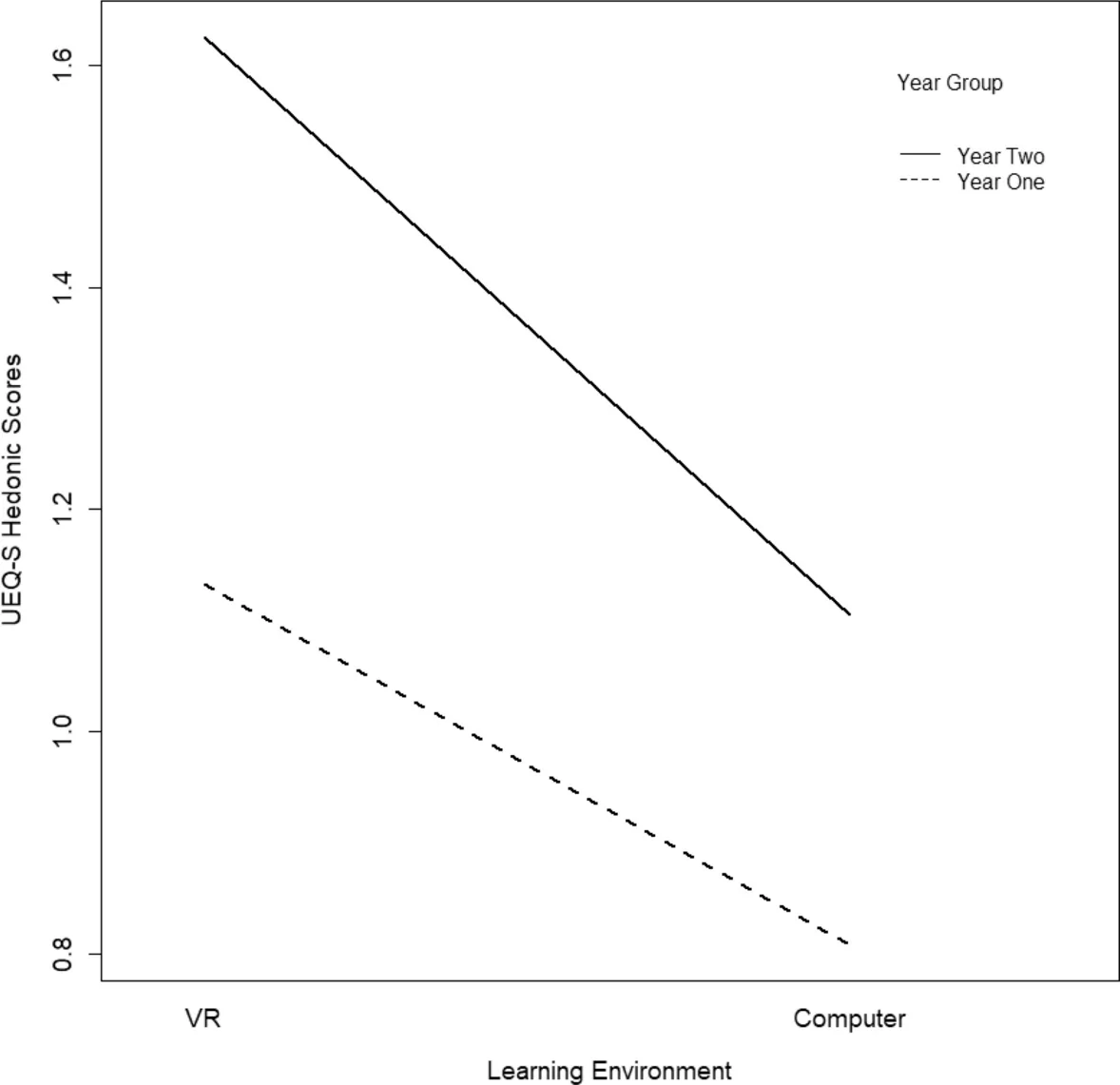
Interaction plot for learning environment and year group on UEQ-S hedonic scores

A boxplot of the UEQ-S hedonic scores across learning environment and year group can be found in Fig. 8. The interaction plots for learning environment and year group on UEQ-S hedonic scores can be found in Fig. 9. A copy of the descriptive statistics for the UEQ-S hedonic scores across the VR and computer conditions split by year group can be found in Table 2.

**Table 2:** Descriptive statistics for UEQ-S hedonic scores in VR and Desktop conditions split by year group

	Mean (± SD)		
	VR UEQ-S Hedonic Score	Computer UEQ-S Hedonic Score	Total
Year 1	1.32 (1.09)	0.81 (1.01)	0.97 (1.05)
Year 2	1.63 (1.17)	1.11 (1.50)	1.36 (1.36)
Total	1.39 (1.14)	0.97 (1.29)	

### Presence

The linear mixed effects model indicated that there was no significant main effect of learning environment on presence scores, F(1, 34) = 0.27, *p* = 0.605, np^2^ < 0.01. The VR learning environment (mean = 4.52, SD = 1.43) did not produce significantly greater presence scores compared to the computer learning environment (mean = 4.46, SD = 1.33).

There was no significant main effect of year group on presence scores, F(1, 43) = 0.30, *p* = 0.588, np^2^ < 0.01. The year one group (mean = 4.40, SD = 1.19) did not produce significantly greater presence scores compared to the year two group (mean = 4.57, SD = 1.54).

Finally, no significant learning environment interaction was found, F(1, 34) = 1.14, *p* = 0.292, np^2^ = 0.03.

**Fig. 10:**
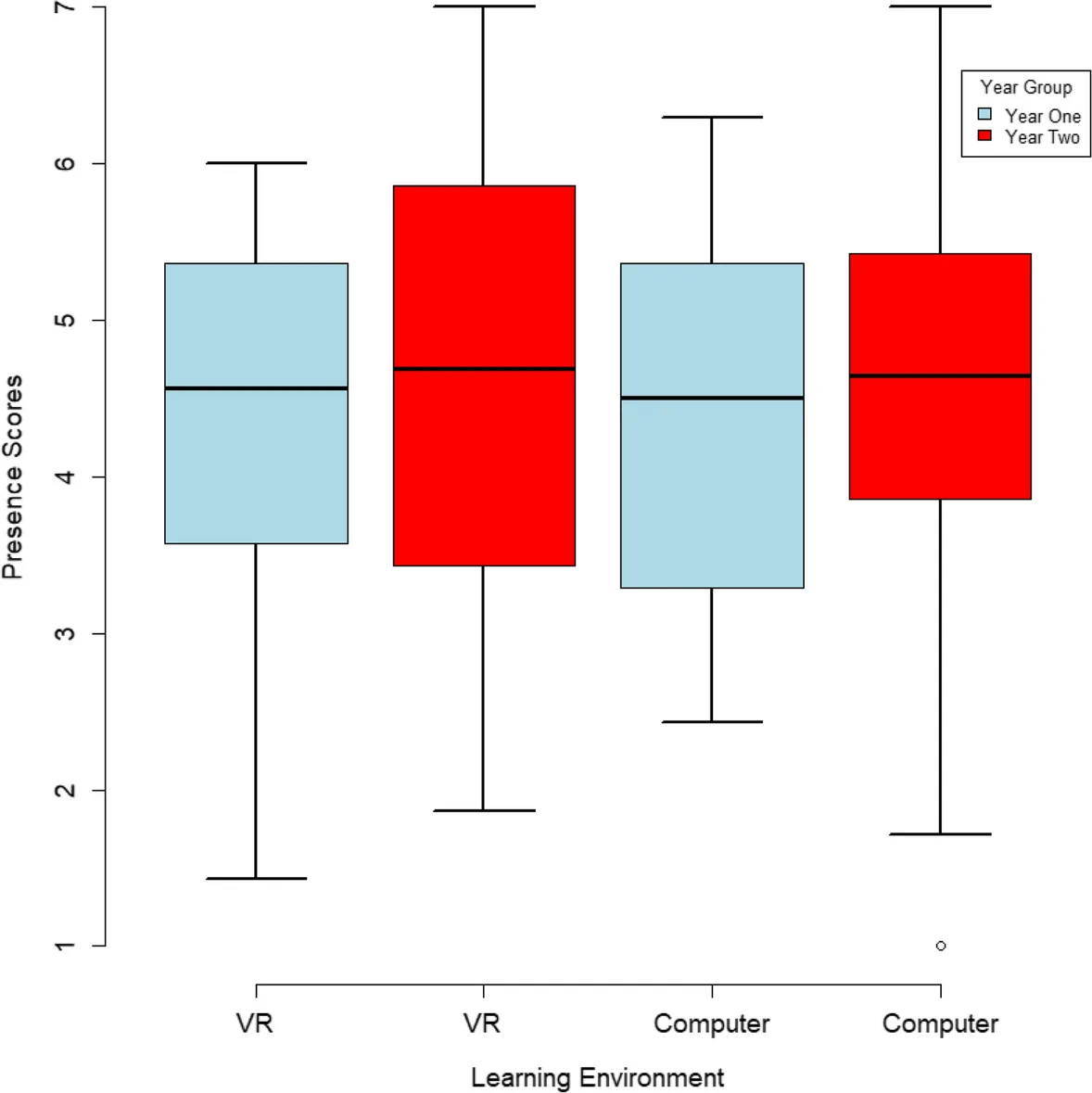
Boxplots comparing presence scores across learning environment and year group

**Fig. 11:**
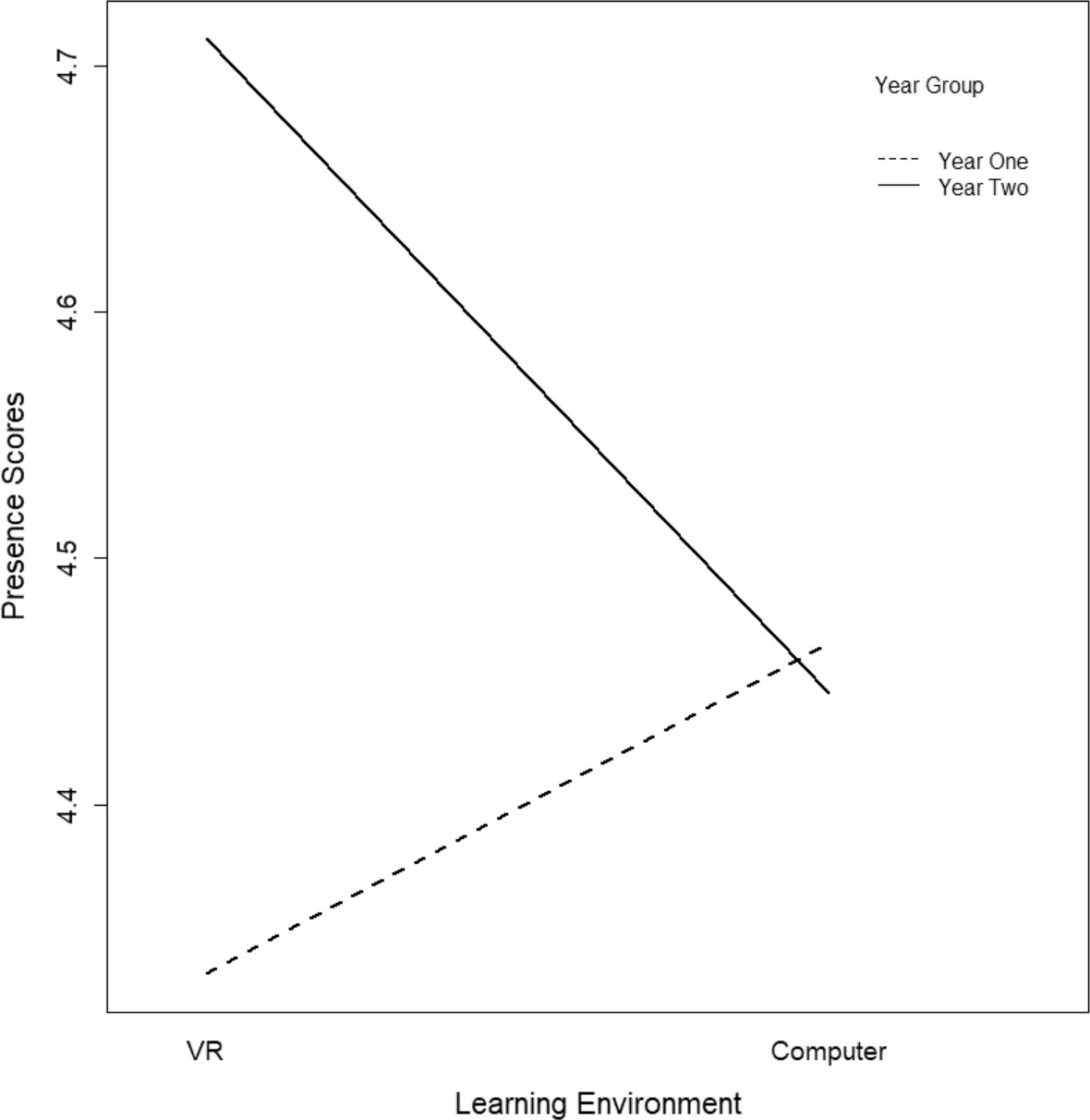
Interaction plot for learning environment and year group on presence scores

A boxplot of the presence scores across learning environment and year group can be found in Fig. 10. The interaction plots for learning environment and year group on presence scores can be found in Fig. 11. A copy of the descriptive statistics for the presence scores across the VR and computer conditions split by year group can be found in Table 3.

**Table 3:** Descriptive Statistics for presence for VR and Desktop conditions split by year group

	Mean (± SD)		
	VR Presence	Computer Presence	Total
Year 1	4.33 (1.30)	4.47 (1.11)	4.40 (1.19)
Year 2	4.71 (1.57)	4.45 (1.54)	4.57 (1.54)
Total	4.52 (1.43)	4.46 (1.33)	

### Comfort with proximity to patient

The linear mixed effects model revealed no statistically significant main effect of learning environment on comfort scores, F(2, 61) = 0.29, *p* = 0.746, np^2^ < 0.01. Neither the VR learning environment (mean = 5.09, SD = 0.93) nor the computer learning environment (mean = 5.05, SD = 0.90) produced significantly greater comfort scores compared to at the beginning of the study (mean = 5.03, SD = 0.80). Descriptive statistics for the comfort scores across the VR and computer conditions split by year group can be found in Table 4.

**Table 4:** Descriptive Statistics for comfort with patient proximity scores split by condition and year group

	Mean (± SD)		
	Pre-Comfort	VR Comfort	Computer Comfort
Year 1	4.82 (0.72)	4.67 (0.75)	4.78 (0.76)
Year 2	5.33 (0.83)	5.55 (0.91)	5.33 (0.97)
Total	5.03 (0.80)	5.09 (0.93)	5.05 (0.90)

However, there was a significant main effect of year group on comfort scores, F(1, 51) = 6.47, *p* = 0.014, np^2^ = 0.11. Post-hoc tests indicated that year two students were significantly more comfortable at all three time points compared to year one students. More specifically, year two students (mean = 5.33, SD = 0.83) were significantly more comfortable with proximity to patients before the study compared to year one students (mean = 4.82, SD = 0.72) (*p* = 0.026). Year two students (mean = 5.55, SD = 0.91) were also significantly more comfortable with proximity to patients following the VR condition compared to year one students (mean = 4.67, SD = 0.75) (*p* = 0.020). Finally, year two students (mean = 5.33, SD = 0.97) were significantly more comfortable with proximity to patients following the computer condition compared to year one students (mean = 4.78, SD = 0.76) (*p* = 0.042).

**Fig. 12:**
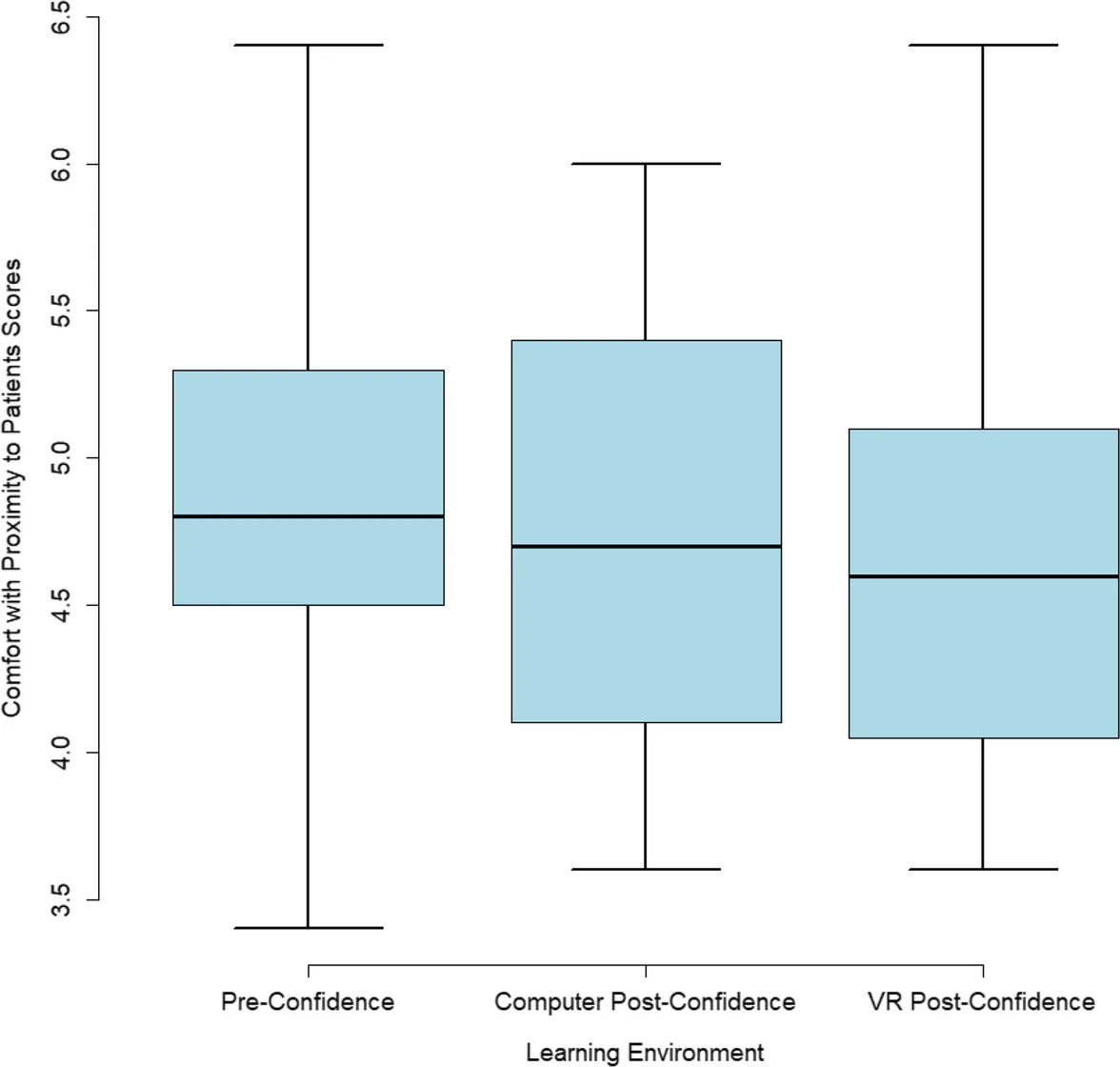
Boxplot comparing comfort with proximity to patients scores across learning environment for year one students

**Fig. 13:**
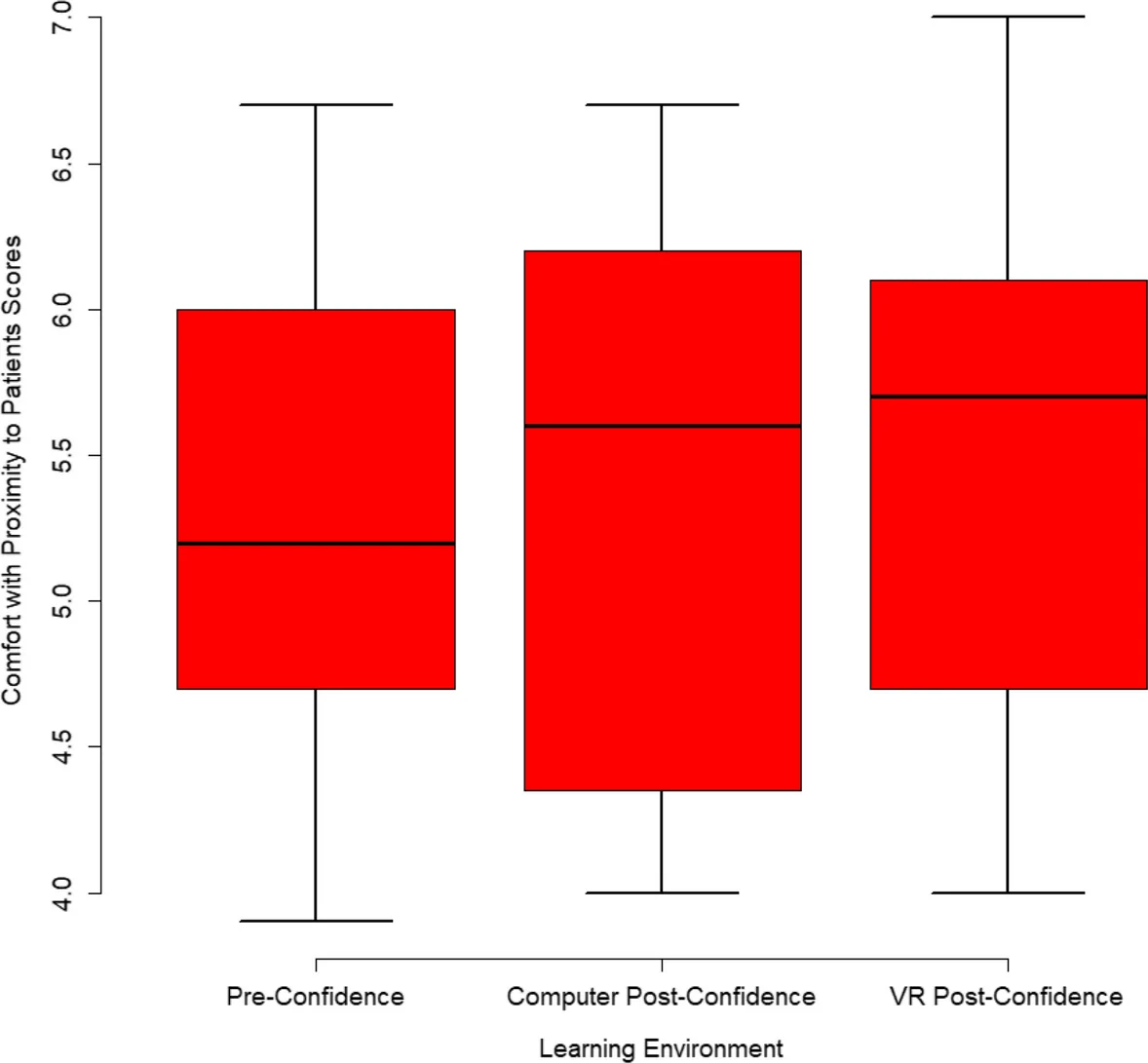
Boxplot comparing comfort with proximity to patients scores across learning environment for year two students

**Fig. 14:**
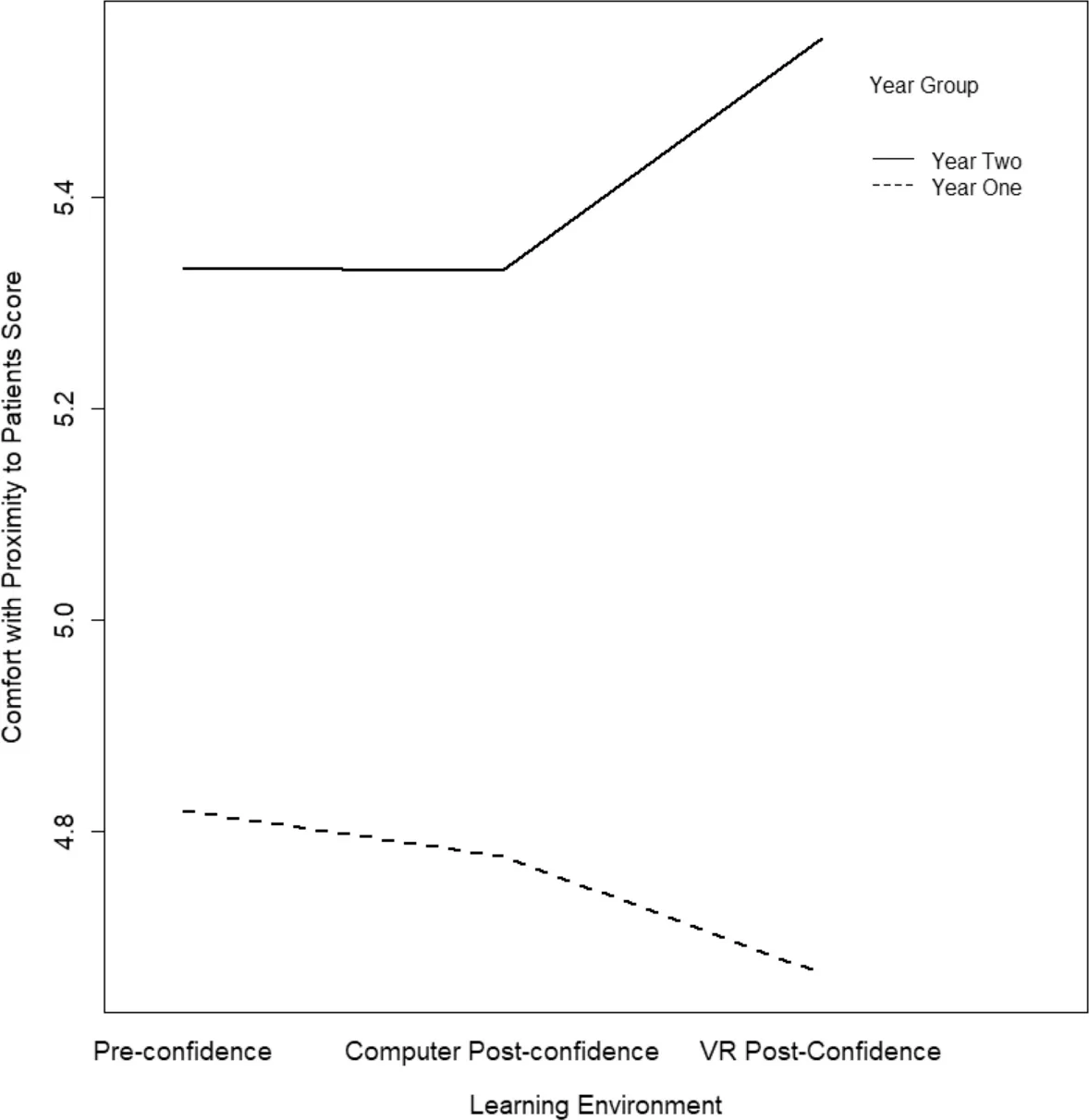
Interaction plot for learning environment and year group on comfort with proximity to patients scores

There was no significant interaction between learning environment and year group on comfort scores, F(2, 61) = 0.12, *p* = 0.887, np^2^ < 0.01 (see Figs. 12, 13 and 14).

### Students in higher education

The first analysis found that 76.9% of participants demonstrated a preference for using a HMD VR over a 2D display, 15.4% had no preference and 7.7% preferred a 2D display.

The second analysis found that in relation to whether the use of VR systems increased interest in teaching content, 87.5% of participants agreed and 12.5% disagreed.

Finally, the third analysis found that in relation to whether students believe that the introduction of VR in education and in the curriculum would improve learning outcomes, 82.6% of students agreed, 13% had no preference and 4.4% disagreed.

## Discussion

This study set out to investigate students’ experiences whilst using a collaborative VR learning environment compared to a desktop computer simulation for orthoptics education. The findings of this study indicated that students perceived the VR simulation to be more enjoyable than the computer-based Strabismus simulator, but less able to help them achieve their goals. The VR was also found to be similar in terms of sense of presence and comfort with proximity to patients. Students also reported having a greater preference for using VR as a form of learning in higher education compared to using a 2D display.

The significant preference for the computer-based Strabismus simulator over the VR simulation could potentially be explained by the technical complexity of the VR system. Students are unlikely to have previous experience with VR, given that only 8% of students have access to a VR headset, whereas 93% have access to a desktop (Student Device Usage Report 2022). As a result, students may have felt more confident navigating and interacting with the desktop-based Strabismus simulator due to its familiarity. In contrast, the VR condition required participants to learn how to operate a head-mounted display, use handheld controllers and navigate the 3D environment within a short space of time before completing the diagnostic task. This additional cognitive demand may have led to a reduction in the perceived usability of the VR simulation.

In addition to device familiarity, the Strabismus simulator has been available for use for the last ten years (American Academy of Ophthalmology n.d.), meaning that there is a significant likelihood of numerous updates having been made based on user feedback over the years. Particularly as the popularity of the simulation is high, with there being currently over 110,000 views (American Academy of Ophthalmology n.d.) at the time of writing this paper. Since the VR Orthoptics simulation has only been available for one year, this study is the first of its kind to receive real world feedback on the VR simulation. Therefore, further development of the VR simulation based on user feedback is likely to result in more positive changes relating to the user experience hedonic score. Overall, these findings indicate that a VR environment can provide an enjoyable and engaging learning environment to practice their diagnostics skills. This perceived usefulness is supported by one participant reporting that the VR learning environment “is good as a form of revision”.

With respect to comfort in proximity to patients, second year students were significantly more comfortable with proximity to real world patients than first year students. Since first year students who participated in this study had not completed their clinical placement, this finding indicates that the placement is likely to have had a positive influence on this aspect of second year students’ training. Whilst the VR and computer simulations did not show a significant improvement in comfort with proximity to patient scores, it suggests that these simulated environments could be used alongside clinical placements to allow students to practice their diagnostics skills. In particular, as users’ scores on the SHEQ questionnaire indicated a preference for using VR in their higher education learning, further research may wish to investigate how VR could be implemented into the Orthoptics university curriculum.

### Applications

Based on the SHEQ and UEQ-S hedonic scores, students indicated that they have a preference for learning in a VR environment over a 2D environment such as that of a computer. This preference is in line with other diagnostics-based fields, such as medicine (Li et al. 2017; Singh et al. 2020) and veterinary (Fejzic et al. 2019), who have already shown movement towards implementing VR for skills training. Whilst the findings of this study do not show a significant difference in sense of presence between learning on a VR and a computer environment, VR is still able to offer a range of benefits such as improving users’ motor skills development (Juliano and Liew 2020). Therefore, universities should consider the implementation of a VR based training approach and how this can be incorporated into the Orthoptics curriculum.

### Limitations

Due to time limitations, students were only allowed to take part in the study for a maximum of 30 min. As the setup of the user in the VR equipment, the explanation of how to use the equipment, troubleshooting, and loading of the simulation all took a significant portion of the available time, students were only able to diagnose one patient in the VR condition. This asymmetry in number of patients between the VR and computer task may have influenced students’ perceptions of the usability and effectiveness of the learning environments, particularly in relation to the UEQ-S pragmatic scores, which assess how well a system supports users in achieving their goals. As a result, the significantly greater pragmatic scores observed in the computer condition may not be solely due to differences in the learning platforms, but also differences in the number of patients students examined in each condition. Future research should equate the number of patients they are exposed to across the learning environments to better isolate this effect on students’ experiences.

Since understanding how to use VR equipment has a steep learning curve (Crogman et al. 2025) the limited time frame of this study may have made VR less appealing compared to the familiarity of a desktop computer. This could potentially explain why significant findings were shown for user experience between the VR and the computer condition. To address this, the authors have two suggestions on how VR user experience could be improved. Firstly, as the novelty of VR HMDs can hinder learning outcomes (Hamilton et al. 2021) students could be made more familiar with the technology before introducing them to the Orthoptics VR simulator. Secondly, as the VR simulation had a total of six virtual patients available (see Appendix E for illustrations of these six patient profiles), each with customisable traits, further research may wish to replicate this study over a longer time frame to assess whether the diagnosis of all six virtual patients could make students more comfortable with using the equipment.

As students only interacted with the learning environments for a short duration, the present study was unable to assess how repeated exposure to VR and desktop simulations alongside traditional teaching methods could influence student’s experiences over time. Future research could integrate these technologies alongside conventional Orthoptics training across a full academic year. This would allow researchers to evaluate how students adapt to the technologies over time and whether the initial novelty and usability challenges associated with VR diminish with continued exposure. Furthermore, incorporating questionnaires or qualitative interviews throughout the learning process could provide a more in depth understanding of students’ experiences whilst using these technologies.

Given that students’ diagnostic abilities were only assessed in groups, there is the possibility of less knowledgeable or quieter students relying on the diagnosis formed by more knowledgeable or outspoken members of the group. This limitation raises questions as to whether the discussed benefits of the simulation apply to the group collectively or on an individual basis. Furthermore, as students were encouraged to communicate throughout the diagnostic task, participants’ perceptions of the learning environments may not have represented entirely independent experiences. Instead, questionnaire responses relating to factors such as usability, enjoyment, and presence may have been influenced by interactions and discussions within the group, introducing potential clustering effects at the group level. Although condition order was counterbalanced across groups, group identifiers and condition order were not retained in the final dataset and therefore could not be incorporated into the statistical models. As a result, the reported standard errors and significance tests should therefore be interpreted with some caution as they may not fully account for group level dependence or order effects. Future research should address this limitation by assessing participants individually, reducing the opportunities for peer influence during the tasks, and recording condition order for statistical analyses.

Large Language Models (LLM) have become increasingly popular in recent years, with their integration being shown in a range of educational areas. This includes within language learning (Liu et al. 2024), coding classrooms (Nie et al. 2025), and the development of students’ understanding of complex concepts (Puente et al. 2024). Integrating an LLM within the Orthoptics simulation would allow students to speak with the virtual patients in real-time, providing them with an opportunity to practice and develop their verbal diagnostic skills. For example, students could be tasked with justifying their diagnosis using key information provided by the virtual patient regarding their background.

A further limitation is that during the VR condition, only one student within each group directly interacted with the VR simulation, while the remaining students observed the simulation on the laptop. As participant role was not recorded, it was not possible to distinguish between headset users and observers during the analyses. As a result, questionnaire responses following the VR condition likely reflected a combination of immersive and observational experiences. This is particularly relevant for measures such as user experience and sense of presence, which may differ depending on whether participants directly interacted with the VR environment. Future studies should ensure that all participants experience the VR simulation individually or record participant role to enable role specific analyses to be conducted.

## Conclusion

This study compared students’ experiences of using a collaborative VR and desktop simulation for Orthoptics education. Students reported significantly higher hedonic user experience when using the VR system, indicating that students perceived it to be a more enjoyable learning environment. Responses to the SHEQ further indicated that students preferred VR over a 2D display as a learning tool and perceived that it could increase interest in educational content within higher education. However, the desktop simulation received significantly higher pragmatic user experience scores, suggesting that it was perceived as better for helping students achieve their learning goals. No significant differences were observed between the learning environments for students’ sense of presence or comfort with patient proximity. These findings suggest that VR has potential as a supplementary training tool within Orthoptics education. VR simulations may provide Orthoptics departments with an additional controlled learning environment that could complement clinical placements and support opportunities for simulated diagnostic practice.

## Data Availability

The datasets generated and analysed during the current study are available on the Open Science Framework (OSF) repository, https://osf.io/fapke/overview?view_only=6d221fb5a6a342ec952cfec770488cd7

## Appendix A

### Comfort with proximity to patients questionnaire

Imagine that you are placed in a room with a patient and asked to perform a procedure that involves a Cover test and Ocular motility skills. To what extent do you agree with the following statements from 1 (Strongly Disagree) to 7 (Strongly Agree).

**Table:**

	Strongly disagree	Disagree	Somewhat disagree	Neither agree nor disagree	Somewhat agree	Agree	Strongly agree
I would feel uncomfortable being close to the patient	O	O	O	O	O	O	O
I would feel comfortable being able to use equipment near the patient’s face	O	O	O	O	O	O	O
I would begin to get anxious as I get closer to the patient	O	O	O	O	O	O	O
I would feel uncomfortable when the patient and I make eye contact	O	O	O	O	O	O	O
I would be confident that I would be able to perform all the steps of the procedure accurately	O	O	O	O	O	O	O
I would doubt that I had made the correct diagnosis	O	O	O	O	O	O	O
I would be confident that I would be able to use the equipment correctly	O	O	O	O	O	O	O
I would be confident that I could perform all the steps of the procedure within an appropriate time	O	O	O	O	O	O	O
I would take too long to come to a correct diagnosis	O	O	O	O	O	O	O
I would be able to use all of the equipment in the correct order	O	O	O	O	O	O	O

## Appendix B

### UEQ-S questionnaire

I found the simulation to be…

**Table:**

obstructive	O	O	O	O	O	O	O	supportive
complicated	O	O	O	O	O	O	O	easy
inefficient	O	O	O	O	O	O	O	efficient
clear	O	O	O	O	O	O	O	confusing
boring	O	O	O	O	O	O	O	exciting
not interesting	O	O	O	O	O	O	O	interesting
conventional	O	O	O	O	O	O	O	inventive
usual	O	O	O	O	O	O	O	leading edge

## Appendix C

### Sense of presence questionnaire

Whilst you were in the learning environment…

How natural did your interactions seem? (1 = not natural – 7 = very natural)

**Table:**

1	2	3	4	5	6	7
|	-	-	-	-	-	|

How much did the visual aspects of the simulation involve you? (1 = not involved – 7 = very involved)

**Table:**

1	2	3	4	5	6	7
|	-	-	-	-	-	|

How natural was the mechanism which controlled movement through the simulation? (1 = not natural – 7 = very natural).

**Table:**

1	2	3	4	5	6	7
|	-	-	-	-	-	|

How compelling was your sense of objects moving through space? (1 = not compelling – 7 = very compelling)

**Table:**

1	2	3	4	5	6	7
|	-	-	-	-	-	|

How much did your experience in the simulation seem consistent with your real world experience? (1 = not consistent – 7 = very consistent)

**Table:**

1	2	3	4	5	6	7
|	-	-	-	-	-	|

How compelling was your sense of moving inside the simulation (1 = not compelling – 7 = very compelling)

**Table:**

1	2	3	4	5	6	7
|	-	-	-	-	-	|

How involved were you in the simulation experience? (1 = not involved – 7 = very involved)

**Table:**

1	2	3	4	5	6	7
|	-	-	-	-	-	|

How natural did your interactions with the application seem? (1 = not natural – 7 = very natural)

**Table:**

1	2	3	4	5	6	7
|	-	-	-	-	-	|

How much did the visual aspects of the application involve you? (1 = not involved – 7 = very involved)

**Table:**

1	2	3	4	5	6	7
|	-	-	-	-	-	|

How natural was the mechanism which controlled movement through the application? (1 = not natural – 7 = very natural)

**Table:**

1	2	3	4	5	6	7
|	-	-	-	-	-	|

How compelling was your sense of human objects being through the application? (1 = not compelling – 7 = very compelling)

**Table:**

1	2	3	4	5	6	7
|	-	-	-	-	-	|

How much did your experiences in the application seem consistent with your real-world experiences? (1 = not consistent – 7 = very consistent)

**Table:**

1	2	3	4	5	6	7
|	-	-	-	-	-	|

How compelling was your sense of moving around inside the application (1 = not compelling – 7 = very compelling)

**Table:**

1	2	3	4	5	6	7
|	-	-	-	-	-	|

How involved were you in the application experience? (1 = not involved – 7 = very involved)

**Table:**

1	2	3	4	5	6	7
|	-	-	-	-	-	|

## Appendix D

### Students in higher education questionnaire (SHEQ)

You are almost at the end of the study. Please answer the following statements

**Table:**

	Completely disagree		Neither agree nor disagree		Completely agree
Interaction with the real people in the real world, whether they are lecturers or students, is necessary	O	O	O	O	O
The visual stimuli provided by VR systems is fascinating to the users	O	O	O	O	O
Stimulation of multiple senses lead to a better understanding of educational content (positive stimulation to the senses consequently lead to more impactful experiences and understanding of educational content)	O	O	O	O	O
The classical evaluation system in education (e.g., exams) does not reflect the real knowledge of the respondents	O	O	O	O	O
People learn better through interaction	O	O	O	O	O
Complete immersion in the virtual world frightens me	O	O	O	O	O
Time passes faster for me while I consume content via VR system compared to consuming content via regular 2D displays	O	O	O	O	O
Introducing virtual reality into the classrooms turns learning into entertainment	O	O	O	O	O
Through the learning process, it’s necessary to apply theoretical knowledge to practical examples in order to master a new skill	O	O	O	O	O
Due to the simulation and experience provided by VR, students will continue to explore and research the educational content	O	O	O	O	O
Virtual reality develops students’ creativity	O	O	O	O	O
Unlike VR, which can provide an interactive experience, classical learning boils down to providing facts only	O	O	O	O	O
While I use a VR system, I am always aware that I’m in a virtual world and that none of it is real	O	O	O	O	O
The group’s shared experiences in a shared environment are important	O	O	O	O	O
The classical evaluation system in education (e.g., exams) reflects the real knowledge of the respondents	O	O	O	O	O
With the help of virtual reality, a student can learn how to react in certain (unknown, dangerous) situations	O	O	O	O	O
With VR, I’m not limited to passively consuming information and images displayed on the screen	O	O	O	O	O
Being able to see and experience the various locations around the world within the classroom provided by VR can inspire and intrigue students	O	O	O	O	O
Virtual environment models teach and train with the same efficiency as reality	O	O	O	O	O
While I use a VR system, I feel like I am present in a virtual world	O	O	O	O	O
Using a VR system would distract students from the educational content	O	O	O	O	O
In the classrooms, there should be mostly interaction between students (the professor only serves as a “guide” to the conversation)	O	O	O	O	O
It’s difficult for me to understand abstract contents and concepts (e.g., energy transfer, and similar) without a visual representation of the same	O	O	O	O	O
Evaluation tailored to the individual, where certain parameters of the respondents are monitored with the help of a VR system, represents a better evaluation system	O	O	O	O	O
I think that my interest in courses and educational content would be higher if interactive content and VR systems were used	O	O	O	O	O
In classrooms, the professor should lead the keynote, i.e., the professor is the main source of information and interaction	O	O	O	O	O
While using VR systems, students can actively learn and participate instead of passively looking at 2D displays	O	O	O	O	O

## Abbreviations

2D: Two Dimensional
3D: Three Dimensional
CPU: Central Processing Unit
GPU: Graphics Processing Unit
HMD: Head Mounted Display
LLM: Large Language Model
OSF: Open Science Framework
CPPQ: Comfort with Proximity to Patients Questionnaire
RAM: Random Access Memory
SHEQ: Students in Higher Education Questionnaire
SD: Standard Deviation
UEQ-S: User Experience Questionnaire Short
VLE: Virtual Learning Environment
VR: Virtual Reality
